# Supplementary material for: Knowledge Translation of Healthcare Research in Saudi Arabia—Implications for Community Health and Primary Care Under the New Saudi Model of Care: A Narrative Review
Source: Healthcare (Basel). 2025 Sep 29;13(19):2469. doi: 10.3390/healthcare13192469 (PMC12523925; doi:10.3390/healthcare13192469)
Supplement: Supplementary file 1 [file healthcare-13-02469-s001.zip › healthcare-3834806-supplementary.pdf]

Table S1: List of the articles included in the analysis, the synthesis of the results, and the discussion.

Section	Title	Reference
**Results: Knowledge translation in healthcare**	Knowledge Translation at the Canadian Institutes of Health Research	1
	Lost in knowledge translation: time for a map?	13
	Models and approaches for building knowledge translation capacity and capability in health services: a scoping review	14
	What is evidence-informed decision-making?	15
	National Institute for Health and Care Excellence: Guidance	16
	About the National Institutes of Health	17
	About the Health and Medical Research Council	18
	The answer is 17 years, what is the question: understanding time lags in translational research	19
	Integrated knowledge translation (IKT) in health care: a scoping review	20
	What is public involvement in research?	21
	What is involvement in research and what does it achieve? Reflections on a pilot study of the personal costs of stroke	22
	Knowledge Translation Planning Template	23
	Preferred Reporting Items for Systematic reviews and Meta-Analyses 2020	24
**Results: Intertwining of community health, primary care, and knowledge translation**	What is a Healthy Community?	25
	Broadening participation in community problem solving: a multidisciplinary model to support collaborative practice and research	26
	HSE Health & Wellbeing Stakeholder Engagement and Communications Plan 2024 – 2027	27
	Community engagement: a health promotion guide for universal health coverage in the hands of the people 2020	28
	Community-based participatory research (CBPR): Towards equitable involvement of community in psychology research	29
	Socio-Ecological Model	30
	WHAT ARE RE-AIM AND PRISM?	31
	Community Planning for Health Assessment: Data & Benchmarks 2025	32
	Guidelines for effective knowledge mobilization 2025	33
	Research and Development. Definitions	34
	Diffusion theory and knowledge dissemination, utilization and integration	35
	Being a knowledge Broker	36
**Results: Situational assessment of health research**	Insights into Saudi Arabia's Research and Innovation Ecosystem	37
	Saudi National Institute of Health: About us	38
	King Faisal Specialist Hospital and Research Center: About us	39
	King Abdullah International Medical Research: About us	40

**and evidence-based practice in Saudi Arabia**	Saudi Health Council: About the Council	41
	Research Development and Innovation Authority: About	42
	About King Abdulaziz City for Science and Technology	43
	About Saudi Food and Drug Authority	44
	King Saud University: Research 2024	45
	King Abdulaziz University : Research and Innovation 2022	46
	About King Salman Center for Disability Research.	47
**Results: Perspectives on knowledge translation in community health and primary care under the new Saudi Model of Care**	Health Sector Transformation Program	5
	Transforming healthcare: Saudi Arabia's vision 2030 healthcare model	12
	New Model of Care Approach at Primary Health Care	48
	Transformation of health care and the new model of care in Saudi Arabia: Kingdom's Vision 2030	49
	The Saudi Model of Care	50
**Discussion**	Methods and status of a comprehensive community-based intervention focusing on non-communicable diseases and the major risk factors in the Kingdom of Saudi Arabia. The Crown Health Project	51
	Healthcare Priorities, Barriers, and Preferences According to a Community Health Needs Assessment in Jazan, Saudi Arabia: A Cross-Sectional Study	52
	Community Health Needs Assessment In Medina City 2023	53
	Community-based participatory research for the assessment of health needs in Najran Health Cluster	54
	Community Health Needs Assessment of Primary Healthcare in Saudi Arabia: A Cross-Sectional Study	55
	Assessment of community health needs in Makkah: A qualitative study	56
	Barriers to Implementing Evidence-Based Practice among Primary Healthcare Nurses in Saudi Arabia: A Cross-Sectional Study	57
	Knowledge, attitude, and practice toward evidence-based medicine among hospital physicians in Qassim Region, Saudi Arabia.	58
